# Supplementary material for: A Second Gamma-Glutamylpolyamine Synthetase, GlnA2, Is Involved in Polyamine Catabolism in Streptomyces coelicolor
Source: Int J Mol Sci. 2022 Mar 29;23(7):3752. doi: 10.3390/ijms23073752 (PMC8998196; doi:10.3390/ijms23073752)
Supplement: Supplementary file 1 [file ijms-23-03752-s001.zip › ijms-1613084-supplementary.pdf]

International Journal of
Molecular Sciences

Supplementary Materials

```
GlnAst          ----------------SAEHVLTMLNEHEVKFVDLRFTDTKGKEQHVTIPAHQVNAEFF-      43
GlnAsc          -------------MFQNADDVKKFIADEDVKFVDVRFCDLPGVMQHFTLPATAFDPD---      44
GlnA2sc         -------------MDKQQEFVIRTLEERDIRFVRLWFTDVLGFLKSVAVAPAELE-QAF-      45
GlnA3sc         MSESDPVPGGRPGEVERATALSGELTGQGVHGVVLAYVDTAGIARVKTVPTAKLAAAAAW      60
                                    :   :  . :: * : : *  *  :  ::    .

GlnAst          EEG--KMF----DGSSIGGW--KGINESDMVLMPDASTAVIDPFFADST--LIIRCDILE      93
GlnAsc          -AE--QAF----DGSSIRGF--QAIHESDMSLRPDLSTARVDPFRRDKT--LNINFFIHD      93
GlnA2sc         DEG--IGF----DGSAIEGF--ARVYESDMIAKPDPSTFQVLPWRAEAPGTARMFCDILM      97
GlnA3sc         GVGMSPVFDTFLADDSIVGTDVLGSPDGDLRLYPDLDRLTMLA---A-------------      104
                       *     ..:* *       :.*:   ** .   :

GlnAst          PGTLQGYDRDPRSIAKRAEDYLRATGIADTVLFGPEPEFFLFDDIRFGASISGSHVAIDD      153
GlnAsc          PITGEQYSRDPRNVAKKAEAYLASTGIADTAFFGPEAEFYVFDSVRFATRENESFYHIDS      153
GlnA2sc         PDGSPSF-ADPRYVLKRALAR--TSDLGFTFYTHPEIEFFLLKDKPVDGS----------      144
GlnA3sc         ---QPGWAWAPVD------------RITQEGAPHPACGRTVLRRIVAGAAERHGITFRAA      149
                      :   *               :       *     ::

GlnAst          IEGAWNSSTKYEGGNKGHRPGVKGGYFPVPPVDSAQDIRSEMCLVMEQMGLVVEAHHHEV      213
GlnAsc          EAGAWNTGAL--EDNRGYKVRYKGGYFPVPPVDHFADLRAEISLELERSGLQVERQHHEV      211
GlnA2sc         ----------------VPTPADNSGYFDHTPQNIGMDFRRQAITMLESMGISVEFSHHE-      187
GlnA3sc         VEVEWVVGRGD-AGGDAFVPAVSGPAYGAARQVELSDCAADLLAALAAQGVDVEQFHPE-      207
                                      ..  :         *   :    :   *: **  * *

GlnAst          ATAGQNEVATRFNTMTKKADEIQIYKYVVHNVAHRFGKTATFMPKPMFGDNGSGMHCHMS      273
GlnAsc          GTAGQAEINYKFNTLLAAADDLQLFKYIVKNVAWKNGKTATFMPKPIFGDNGSGMHVHQS      271
GlnA2sc         GAPGQQEIDLRYADALSTADNVMTFRLVMKQVALEQGLQATFMPKPFSEYPGSGMHTHLS      247
GlnA3sc         YAAGQFEVSVGALGPVAAADHSVLVRQTIRAVSARHGLRVSFAPAVLGQGVGNGGHLHLS      267
                 : ** *:          **.    :  :: *: . *  .:* *  :    *.* * * *

GlnAst          LAKNGTNLF-SGDKYAGLSEQALYYIGGVIKHAKAINALANPTTNSYKRLVPG-------      325
GlnAsc          LWSGGEPLFYDEQGYAGLSDTARYYIGGILKHAPSLLAFTNPTVNSYHRLVPG-------      324
GlnA2sc         LFEGDRNAFYESGAEYQLSKVGRSFIAGLLRHAAEISAVTNQWVNSYKRIWGGTERTAGA      307
GlnA3sc         AWRDGTNLHAGGTARCGMTAEAESFVAGVLGHLPALTALTAPSPASRLRLRPS-------      320
                   ..   .        ::  . ::.*:: *   : *.:     *  *:  .

GlnAst          -YEAPVMLAYSARNRSASIRIPVVA-S--PKARRIEVRFPDPAANPYLCFAALLMAGLDG      381
GlnAsc          -FEAPVNLVYSQRNRSAAMRIPITGSN--PKAKRVEFRAPDASGNPYLAFSALLLAGLDG      381
GlnA2sc         GGEAPSYICWGHNNRSALVRVPMYKPG-KTGSARVEVRSIDSGANPYLTYAVLLAAGLKG      366
GlnA3sc         -QWAGVFTAWGRETREAALRIVTGTAGIRDRAANLEVKPVDLAANPYLALASVIAAGLDG      379
                   *     :. ..*.* :*:     .    : .:*.:  * ..****  : :: ***.*

GlnAst          IKNKIHPGEPMDKNLYDLPPEEAK--EIPQVAGSLEEALNALDLDREFLKA-----GGVF      434
GlnAsc          IKNKIEPAEPIDKDLYELAPEEHA--NVAQVPTSLGAVLDRLEADHEFLLQ-----GDVF      434
GlnA2sc         IEEGYELPPGAEDDVWALSDAERRALGIEPLPQNLGEALALMERSDLVAETLGEHVFDFF      426
GlnA3sc         LASSAPLPEEITGDPARLDPAAAAARGVRRLPVTLTESVAAFRTDGVLREALGPVLADAV      439
                : .          :   *         :  :  .*   :  :  .  .         . .

GlnAst          TDEAIDAYIALRREE-DDRVRMTPHPVEFELYYSV---      468
GlnAsc          TPDLIETWIDFKRANEIAPLQLRPHPHEFEMYFDV---      469
GlnA2sc         LRNKRQEWEEYRSQ-----------VTAFELRKSLPVL      453
GlnA3sc         IAVRLGEAGSVEG------LDDDGVAAAYRWKY-----      466
```

Figure S1. Identification of the conserved residues of *S. coelicolor* GlnA$_{st}$, GlnA2$_{st}$ and GlnA3$_{st}$ proteins. GlnA$_{st}$ represents to the GS protein sequence from *S. typhimurium*. Conserved amino acids representing the consensus motif of GlnA$_{st}$ and GlnA$_{sc}$ are marked in orange, while alternatives in GlnA2 and GlnA3 in red.

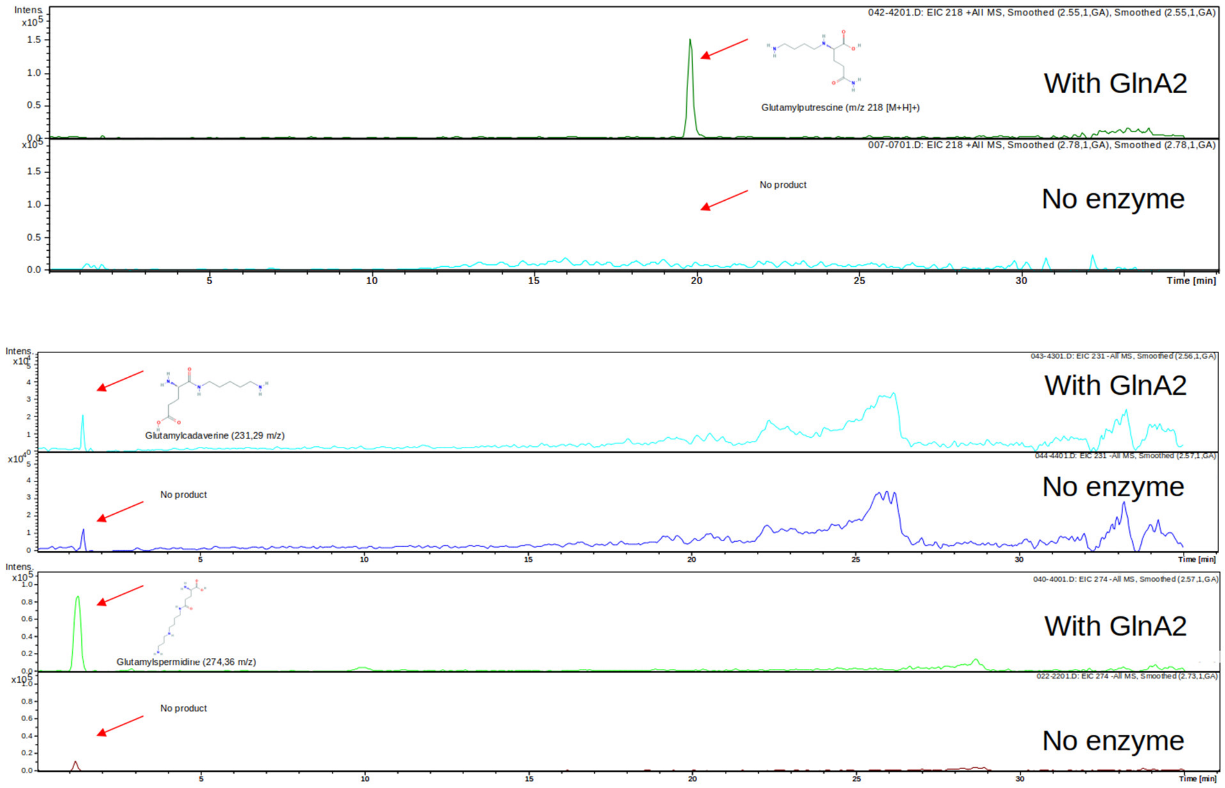

Figure S2. HPLC/ESI-MS analysis of the glutamylated reaction product generated by GlnA2 in an *in vitro* assay (full chromatogramm): top for gamma-glutamylputrescine; middle for gamma-glutamylcadaverine; bottom for gamma-glutamylspermidine. Two samples were analyzed in the MS positive mode (EIC – extracted ion chromatogram): on each chromatogramm reaction mixtures corespond to samples without addition of GlnA2 (below) and with addition of GlnA2 (above). Extracted ion chromatograms for the GlnA2 reaction product corresponding to gamma-glutamylputrescine, gamma-glutamylcadaverine and gamma-glutamylspermidine with charge to mass ratio of 218 m/z, 231 m/z and 274 m/z, respectively, are shown.

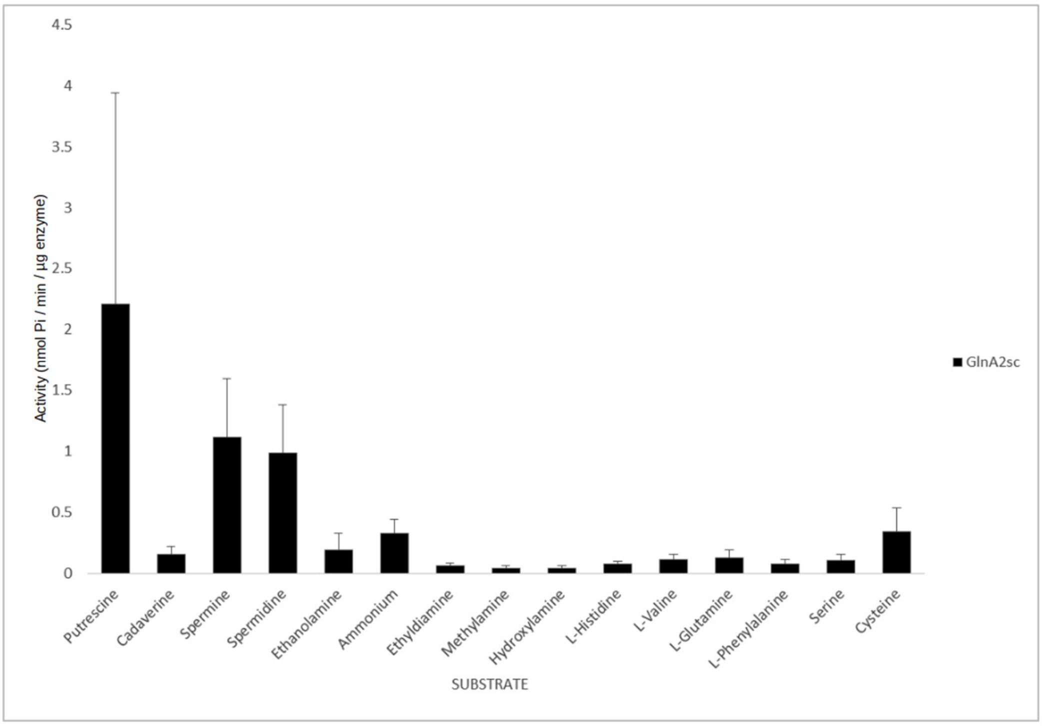

Figure S3. Effect of different nitrogen containing substrates on the activity of GlnA2. All substrates were at a concentration of 50 mM. The mean value of n = 6 biological replicates from different cultures with n = 3 technical replicates each with standard error is shown.

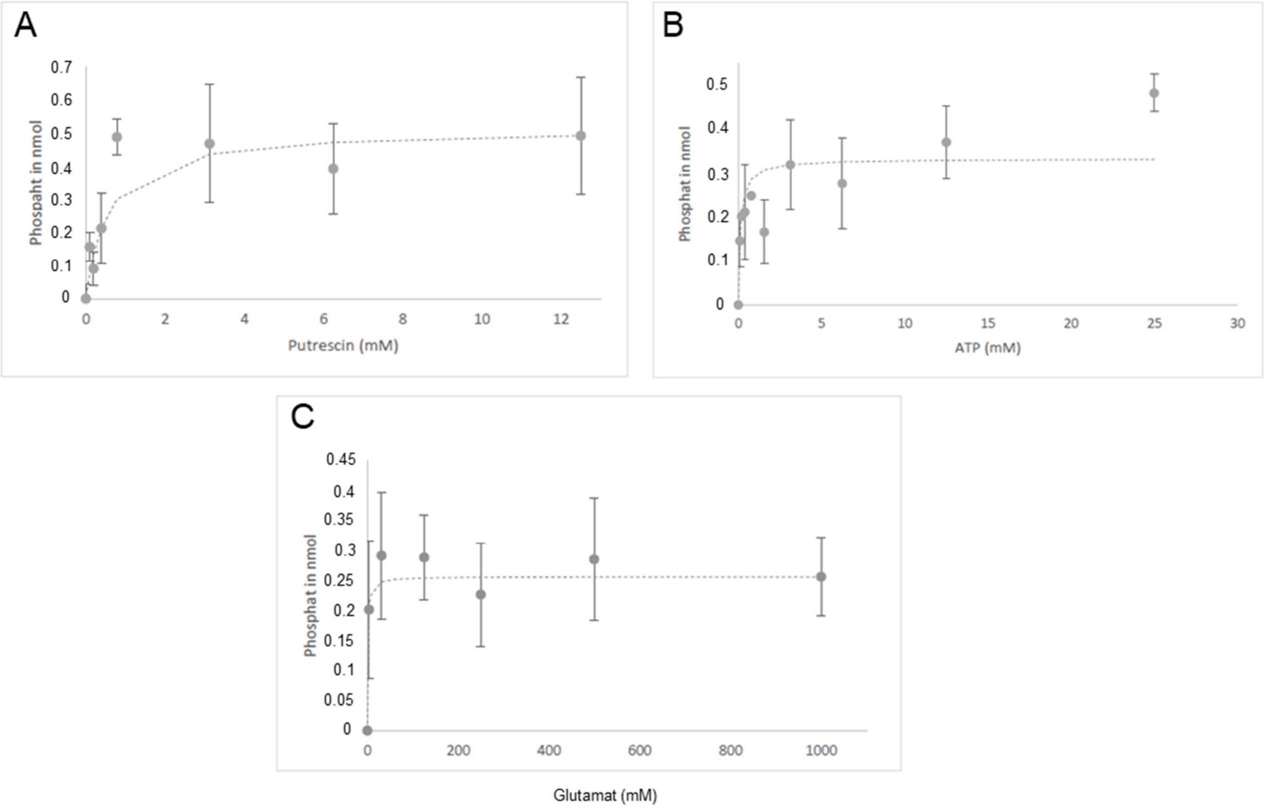

Figure S4. Activity of GlnA2 with different concentrations of putrescine (**A**), ATP (**B**), and glutamate (**C**) as well as in the presence of MSO (D). A nonlinear regression (solid black line, **A**,**B**,**C**) was made using a least-squares fit of n = 3 data sets (grey bullets) and assuming the Michaelis-Menten model.

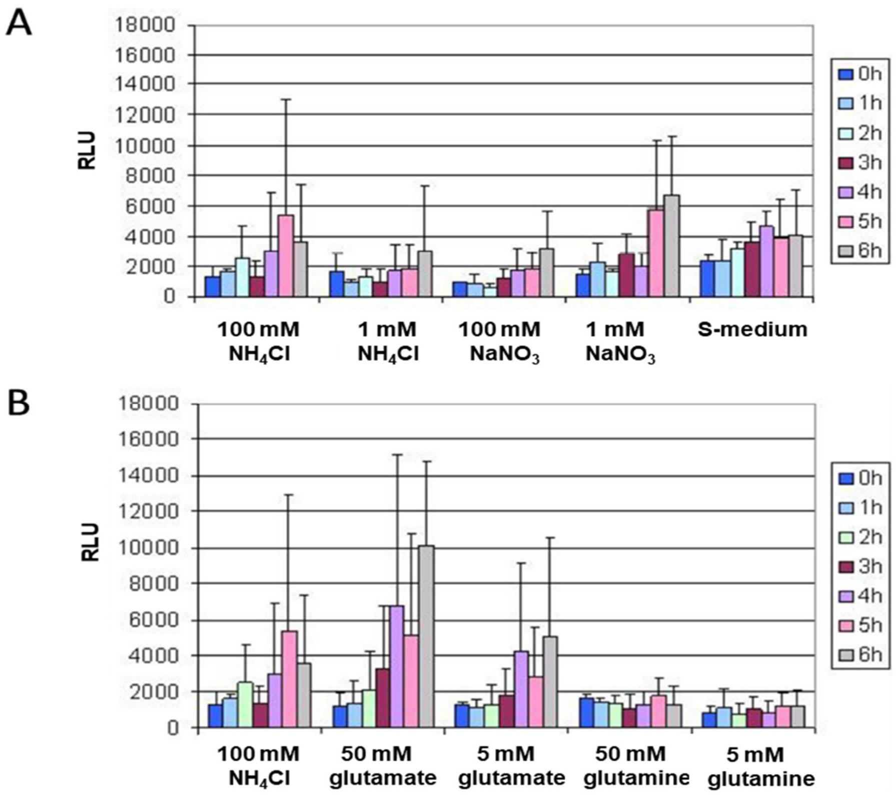

Figure S5. Luciferase-based reporter gene analysis in *S. coelicolor* M145 under different nitrogen conditions. The promoterless genes *luxAB* were integrated under the control of the *glnA2* promoter in the *S. coelicolor* M145 genome. The cells were incubated in a pre-culture complex S-medium, transferred to the main-culture defined Evans medium with respective nitrogen sources, gathered and were shaking for 6 hours in a 96well plate. Samples were controlled every hour. The luminescence expressed as relative light unit (RLU) after incubation in (**A**) S-medium and Evans medium with 1 mM or 100 mM NH_4Cl or $NaNO_3$ and (**B**) 5 mM or 50 mM glutamate or glutamine were determined.

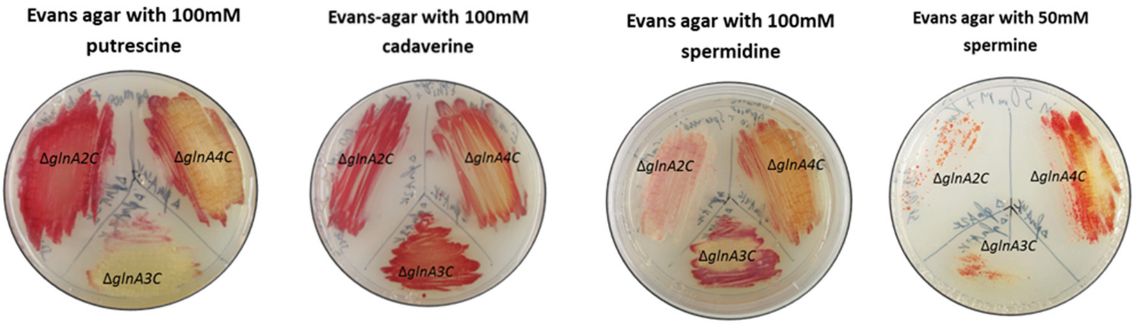

Figure S6. Phenotypic analysis of the parental strain and the complemented *glnA2* (*ΔglnA2C*), *glnA3* (*ΔglnA3C*) and *glnA4* (*ΔglnA4C*) mutants on defined Evans-agar supplemented with polyamines.

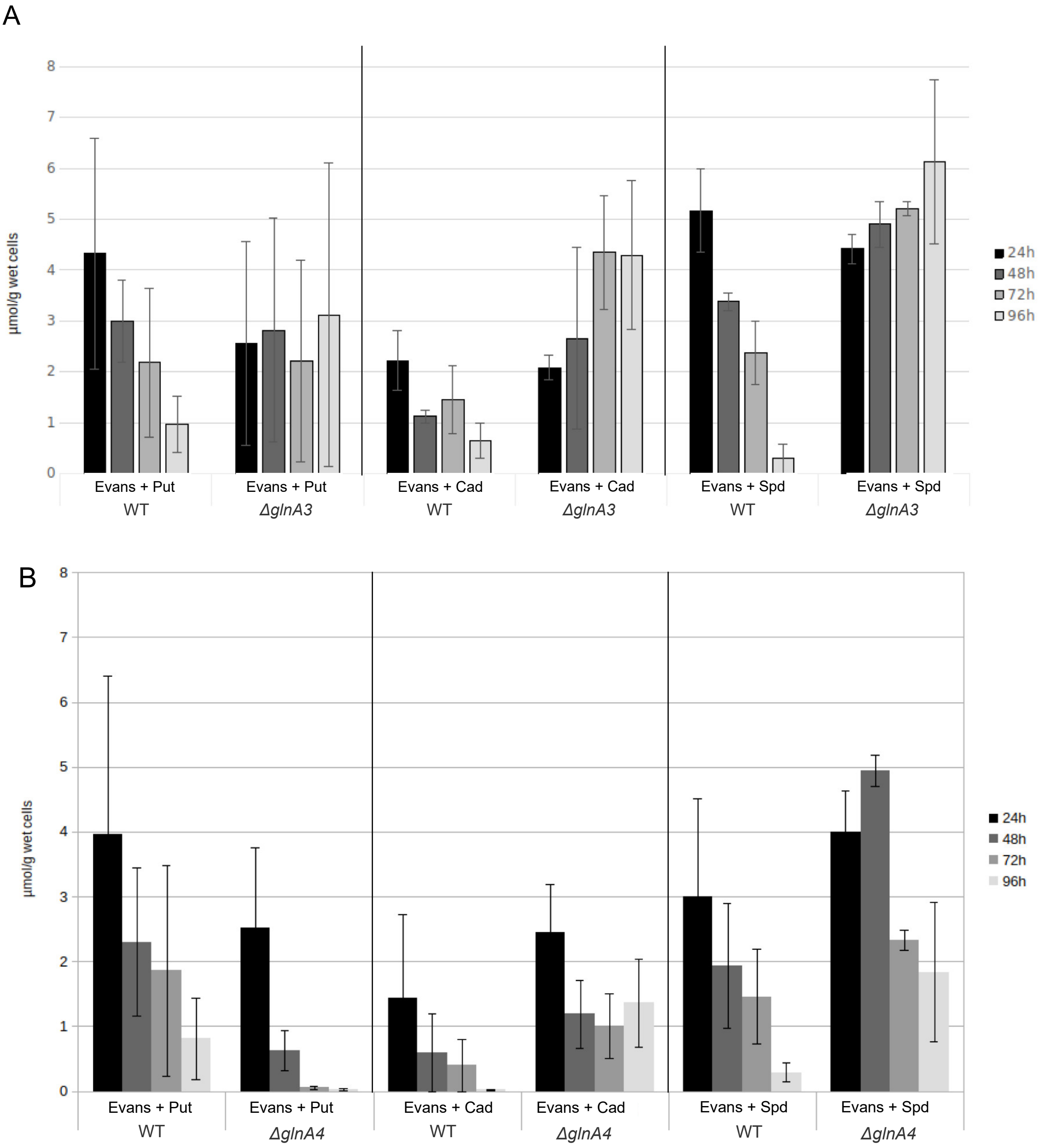

Figure S7. Intracellular polyamine concentration in *S. coelicolor* strains. The polyamine level of the parental strain *S. coelicolor* M145 (WT) and the *ΔglnA3* mutant (**A**) or the *ΔglnA4* mutant (**B**) was monitored in samples taken after 24, 48, 72 and 96 h of cultivation in defined Evans medium supplemented with polyamines (Put – putrescine; Cad – cadaverine or Spd – spermidine, 25 mM of each) as a sole nitrogen source. The mean value of three biological replicates was calculated in μmol per 1 g of wet cells. Error bars indicate standard error of tree (**A**) or two (**B**) biological replicates.

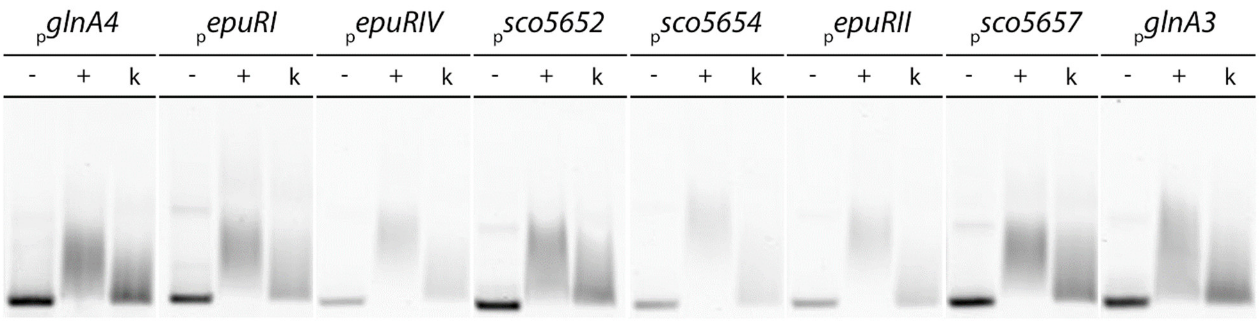

Figure S8. Band shift assays performed with EpuRI. 1 ng fluorescence-labeled promoter regions of polyamine associated genes were incubated without (-) or with (+) 2 µg His-EpuRI. As a control 1000-fold amount of specific unlabeled DNA fragments (K) was added.

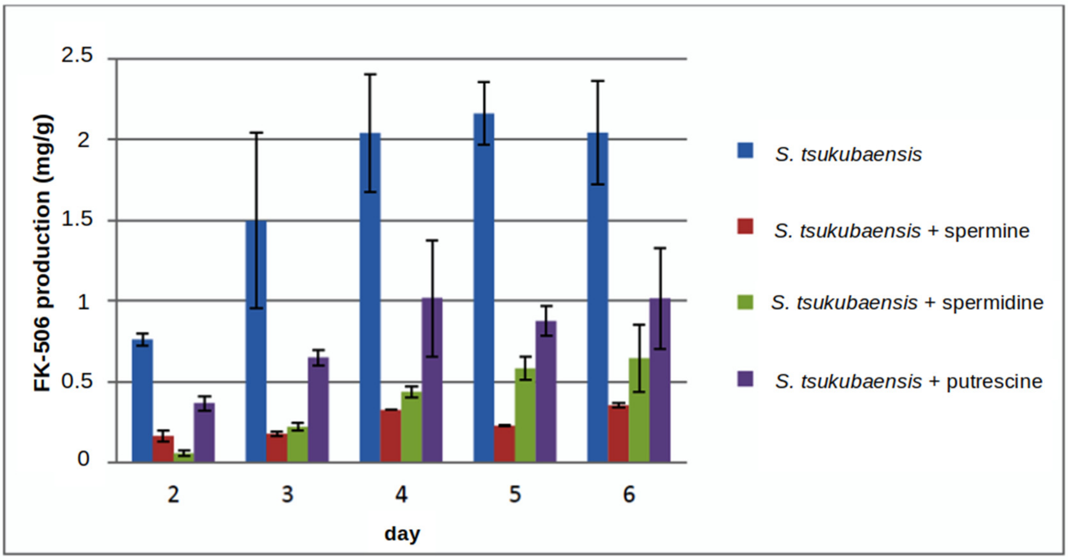

Figure S9. FK-506 production test and biomass estimation in *S. tsukubaensis*. The wild type has been incubated in MG medium with external addition of polyamines putrescine, spermidine or spermine. The specific FK-506 yield in mg / g at the various times (day 2 to day 6) with standard deviation is plotted in the bar diagram.

Table S1. RNAseq analysis results combined as a list with the selection of *S. coelicolor* genes with increased expression in the presence of polyamines compared to the culture with ammonium. Data are expressed as log change of expression compared to the control with ammonium.

Gene	Function	Cadaverine	Putrescine	Spermidine	Spermine
SCO5584	nitrogen regulatory protein P-II 1 | (RefSeq) glnB	4.83	-	-	-
SCO5679	aldehyde dehydrogenase, aldehyde dehydrogenase (NAD+)	3.00	2.91	2.35	-
SCO4159	transcriptional regulator GlnR	2.65	-	-	-

Table S2. Oligonucleotides used in this study.

Oligonucleotides	Sequences 5´-3´	Reference
glnA2-pRSETB-C-Fw	ACATATGGACAAGCAGCAGGAGTTC	This work
glnA2-pRSETB-C-Rev	AAGCTTCTAATGATGATGATGATGATGCAGCACCGG-CAGCGACTT	This work
glnA2-pRSETB-N-Fw	ACCATGGAAGACAAGCAGCAGGAGTTC	This work
glnA2-pRSETB-N-Rev	AAAGCTTCTACAGCACCGGCAGCGA	This work
glnA2-up-fw	AGCCAGTGGCGATAAGCCGTGAGGTCCGCTGATC	This work
glnA2-up-rev	AGCCAGTGGCGATAAGCATCTTCCGCTTCCCCAT	This work
glnA3-up-fw	AGCCAGTGGCGATAAGGCCGTCACCGTCATGGGC	This work
glnA3-up-rev	AGCCAGTGGCGATAAGCACGTGTCGGCTCCTTCG	This work
glnA4-up-fw	AGCCAGTGGCGATAAGCGCAGCGTGACGCGGCTG	This work
glnA4-up-rev	AGCCAGTGGCGATAAGCATAAGGTATTGCCGGGA	This work
rt-hrdB-fw	CAAGCTGGCGAACTCCGACAAG	This work
rt-hrdB-rev	CAGGTGGCGTACGTGGAGAAC	This work
rt-glnA2-fw	CGGCCAGCAGGAGATCGAC	This work
rt-glnA2-rev	CAGGCCCGCGATGAAGGAG	This work
glnA2-del-AB´-Fw	GGGAATTCACCTCACGCACGCCGTCT	This work
glnA2-del-AB´-Rev	CCAGATCTCATCTTCCGCTTCCCCAT	This work
glnA2-del-BC-Fw	GGGGATCCTAGGGCGGCTGTGCCGGCCC	This work
glnA2-del-BC-Rev	AAAAGCTTCGTCTACGCCGTCGCCTTCA	This work
glnA2-Apra-Mut-fw	AGAATTCGACAGCACGATCGGGTCC	This work
glnA2-Apra-Mut-rev	AAGCTTTCGTAGCCCTCCTCGATGCC	This work
glnA2-150bp-up	TGGCCTCTACGCTGCGGT	This work

glnA2-150bp-down	AGAACCGGCCCGAGGACT	This work
glnA2-350bp-int	AGGTGAAGCCCAGGTCGGA	This work
glnA2-1000bp-int	CGAGGTCCGCTCCATCGA	This work
glnE-150bp-up	TCCCTTCCTCGGCTGGTACA	This work
glnE-150bp-down	GACGGCGGAGGGTGTGGTTG	This work
glnE-500bp-int	GCGGTCTCGGCGACGTCGAT	This work
glnE-2500bp-int	TGATCGACCCCCTGCGCTAC	This work
glnE-up-fw	AGCCAGTGGCGATAAGACCGGGGCGTGGTCCTCG	This work
glnE-up-rev	AGCCAGTGGCGATAAGCATCTCGGCCTCCTGTCG	This work
lux-glnA2-fw	AGGATCCGTCCCGGCCCGATCCCCG	This work
lux-glnA2-rev	ATCTAGAATGTCGCGCTCCTCCAGC	This work
5652EMSAf	AGCCAGTGGCGATAAGCACCACGGACGCCCTG	This work
5652EMSAr	AGCCAGTGGCGATAAGCATGGGGCCTCTTCCGGT	This work
5654EMSAf	AGCCAGTGGCGATAAGCAGATCCTGCGCGCCACG	This work
5654EMSAr	AGCCAGTGGCGATAAGCATGGGCGTACACCTCGG	This work
5656EMSAf	AGCCAGTGGCGATAAGTCCGTCCGTCGGCGGCGTCC	This work
5656EMSAr	AGCCAGTGGCGATAAGCACGGCTTCACTGTGCACG	This work
5657EMSAf	AGCCAGTGGCGATAAGCGGCGTCCAACTGGGGAC	This work

5657EMSAr	AGCCAGTGGCGATAAGCACTGCCGGCCCTCCAAG	This work
5658EMSAf	AGCCAGTGGCGATAAGCTACGGCAAGGACCTGTCG	This work
5658EMSAr	AGCCAGTGGCGATAAGCATGGCACGAGGATCCCGT	This work
5666EMSAf	AGCCAGTGGCGATAAGGCTGTCCCGCGCCTCCGGG	This work
5666EMSAr	AGCCAGTGGCGATAAGCATGTCGGCTCTCCTCGGT	This work
5667EMSAf	AGCCAGTGGCGATAAGGGATACAAGGCGTCCGGGT	This work
5667EMSAr	AGCCAGTGGCGATAAGCATGGCGGCCACTTGGGC	This work
5676EMSAf	AGCCAGTGGCGATAAGCCTTGCCCCCTGACCTGCCG	This work
5676EMSAr	AGCCAGTGGCGATAAGCATGGGGGGCTCCTGGGG	This work
5977EMSAf	AGCCAGTGGCGATAAGCGGGCTCGACTCCCTGGA	This work
5977EMSAr	AGCCAGTGGCGATAAGCATTGCGGGTGGTGCTCGT	This work
6960EMSAf	AGCCAGTGGCGATAAGCCGAGTTCCACCACCTGG	This work
6960EMSAr	AGCCAGTGGCGATAAGCATCGCTTGATCATACTTTCAGCC	This work
6963EMSAf	AGCCAGTGGCGATAAGCCGGCGGGTCCGGCCCGTT	This work
6963EMSAr	AGCCAGTGGCGATAAGCATCGCGGGATGCCTCCTG	This work
1614EMSAf	AGCCAGTGGCGATAAGTCGAGGAAGAACCGGGCG	This work
1614EMSAr	AGCCAGTGGCGATAAGCACGCTCCCGGCACTCC	This work
1615EMSAf	AGCCAGTGGCGATAAGCCTGAACGACCTGCTGG	This work

1615EMSAr	AGCCAGTGGCGATAAGCATCCGTCCTCCAGGTGA	This work
EMSA1616F	AGCCAGTGGCGATAAGTACCAGTTGGGCCCGAGAT	This work
EMSA1616R	AGCCAGTGGCGATAAGCTGCCCATAACCCCAGCTT	This work
EMSA1617F	AGCCAGTGGCGATAAGCTGCCCATAACCCCAGCTT	This work
EMSA1617R	AGCCAGTGGCGATAAGTACCAGTTGGGCCCGAGAT	This work
EMSA1619F2	AGCCAGTGGCGATAAGAAGGCCATCCGCGCCTTGAA	This work
EMSA1619R2	AGCCAGTGGCGATAAGTTTGTGGTCGCCCATCGCT	This work
